# Complete Genome Sequence of Moraxella osloensis Strain YV1, Isolated from an Australian Wastewater Treatment Plant

**DOI:** 10.1128/MRA.00030-20

**Published:** 2020-04-09

**Authors:** Steven Batinovic, Daniel T. F. Rice, Robert J. Seviour, Steve Petrovski

**Affiliations:** a Department of Physiology, Anatomy, and Microbiology, La Trobe University, Bundoora, Australia; University of Delaware

## Abstract

We report the complete genome sequence of Moraxella osloensis strain YV1, which was isolated from a wastewater treatment plant in Australia. The YV1 genome comprises a 2,615,801-bp chromosome and four plasmids. Moraxella osloensis strain YV1 displays the distinctive morphology of Eikelboom morphotype 1863.

## ANNOUNCEMENT

**FIG 1:**
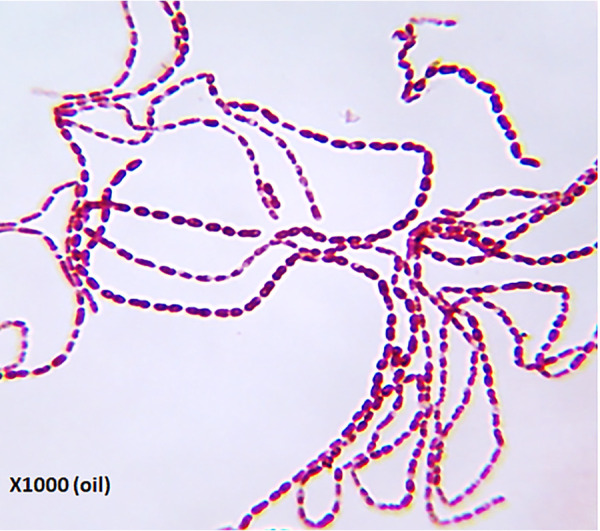
Gram staining and morphology of *Moraxella osloensis* strain YV1.

Moraxella osloensis is a Gram-negative organism belonging to the family *Moraxellaceae*. It has been implicated in causing malodors in laundries (1, 2), is an opportunistic pathogen (3), and appears as a filamentous bacterium resembling the Eikelboom filamentous morphology (Fig. 1) of type 1863 (4), causing bulking in wastewater treatment plants.

Here, we report the complete genome sequence of M. osloensis strain YV1, which was isolated from a wastewater treatment plant in Victoria, Australia. Although members of the aerobic genus Moraxella are considered to grow characteristically as coccobacilli in pairs, this strain was isolated by micromanipulation, using a sterile glass hook to isolate a single filament from the biomass onto the surface of agar, as described by Blackall (5). The isolate was grown on R2A agar for 24 h at 28°C. The complete sequence of its 16S rRNA gene (positions 27 to 1492) showed 99.87% BLASTn identity with respect to type strain Moraxella osloensis CCUG 350. Genomic DNA from a liquid culture was extracted using the Wizard genomic DNA purification kit (Promega) and sequenced by Illumina short-read and Oxford Nanopore long-read sequencing technologies. Short-read libraries for Illumina sequencing were prepared using the NEBNext Ultra II for DNA library preparation kit (NEB), and paired-end sequencing was performed on an Illumina MiSeq v3 system (600 cycles), generating 1,984,246 sequences in pairs. Long-read libraries generated with the Oxford Nanopore MinION system (R9.4.1 SpotON flow cell) were prepared using the ligation sequencing kit (LSK108) with native barcoding expansion (NBD104), generating 18,802 sequences with an average length of 7.3 Mb. Short-read data were filtered using Trim Galore v0.6.4 (6) with the default settings (Q scores of ≥20, with automatic adapter detection), and long-read data were trimmed to remove sequence reads of ≤2,000 bp with CLC Genomics Workbench v9 (Qiagen). The genome was hybrid assembled using Unicycler v0.4.8 (7), generating 5 circular contigs with 166-fold average genome coverage. Except where noted, program defaults were used.

The YV1 genome comprises a 2,615,801-bp chromosome (G+C content of 43.5 mol%) and four plasmids (p1 to p4) of 127,800 bp, 34,071 bp, 26,254 bp, and 15,781 bp, with G+C contents of 41.2%, 41.5%, 39.4%, and 39.4%, respectively. When the YV1 genome was annotated using the NCBI Prokaryotic Genome Annotation Pipeline (PGAP) v4.10 (8), the chromosome contained 2,322 predicted coding sequences (CDSs), 47 tRNA genes, and 4 rRNA operons. The plasmids p1 to p4 consist of 125, 30, 30, and 19 predicted CDSs, respectively. A type I-F CRISPR-Cas module containing 114 CRISPR spacers was detected with CRISPRCasFinder v1.1.2 (9). No complete prophages were detected in the genome using PHASTER (10). Genomic indices, including average nucleotide identity (ANI) (JSpeciesWS v3.4.0 [11]), average amino acid identity (AAI) (CompareM v0.1.0 [https://github.com/dparks1134/CompareM]), and digital DNA-DNA hybridization (DDH) (Genome-to-Genome Distance Calculator v2.1 [12]) between YV1 and the type strain M. osloensis CCUG 350 (GenBank accession number CP014234), were determined to be 94.7%, 95.7%, and 59.9%, respectively, which are at the low end of suggested species delineation thresholds (95 to 96% for ANI and AAI [13, 14] and 70% for DDH [15]). A whole-genome alignment of YV1 and type strain CCUG 350, performed with the Mauve v1.1.1 plugin (16) in Geneious Prime v2020.0.5 using the progressiveMauve algorithm and visualized using genoPlotR v0.8.9 (17), revealed that a large inversion event had occurred between positions at 0.33 and 2.29 Mb of the YV1 genome.

### Data availability.

The M. osloensis strain YV1 genome sequence was deposited in GenBank under the accession numbers CP047226 (chromosome) and CP047227, CP047228, CP047229, and CP047230 (plasmids). Raw data are accessible under BioProject accession number PRJNA598073.
